# Clinical trials information mediator (CTIM)

**DOI:** 10.1186/2043-9113-5-S1-S7

**Published:** 2015-05-22

**Authors:** Benjamin Braasch, Töresin Karakoyun

**Affiliations:** 1 Clinical Research Informatics, Heinrich-Heine University Düsseldorf, 40225 Düsseldorf, Germany

## Characterisation

Service / tool, search engine, clinical trials, publications, trials registry, biosamples, open source.

## Tool description

Developed by the BioMedBridges project, the Clinical Trials Information Mediator (CTIM) integrates different kinds of information derived from variable sources and supports researchers, especially those working in the personalized medicine area, by providing insights into the effects of drugs based on clinical trials data, genomic information and published results. CTIM is one of the tools provided by the BioMedBridges project to enable building data-bridges between different biomedical research infrastructures.

**Figure 1:**
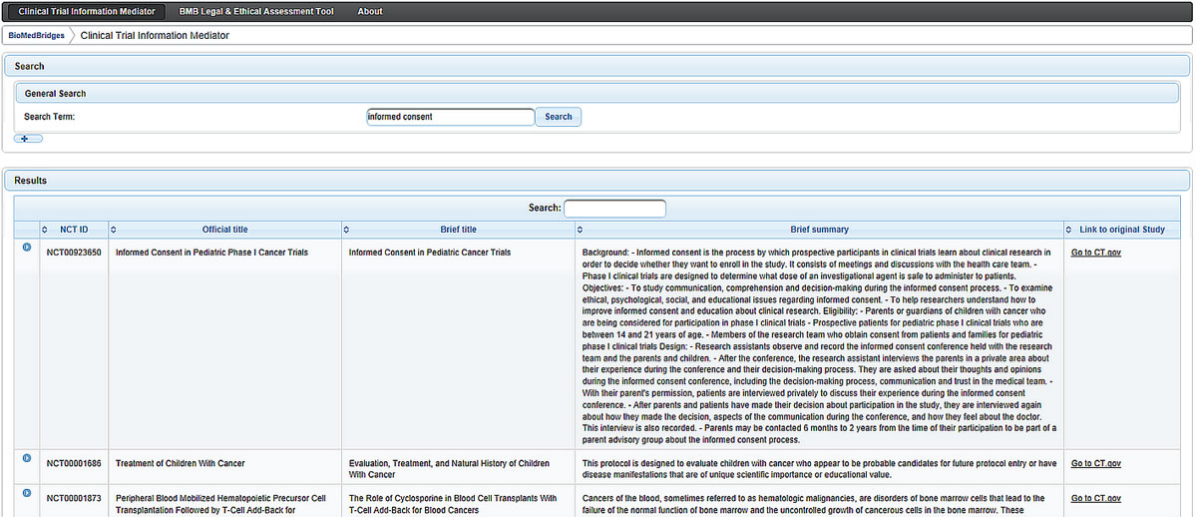
CTIM user interface. Results of a query for “informed consent” is shown. Left side indicates NCT numbers; the right side offers links to ClinicalTrials.gov.

CTIM is still in development and the first release candidate offers clinical trials information that is provided by the international study registry ClinicalTrials.gov over a graphical user interface and a RESTful web-service (Figure 1). The tool is a free to use web service and it is planned to enable the integration of information from different biomedical databases that were not linked before. Though, the actual release offers only access to clinical trials data (ClinicalTrials.gov), links to biomedical databases are being established for later releases. CTIM has been developed with Java Technology. Deployed as a portlet for Liferay Portal, CTIM is accessible over any compatible web browser. The RESTful web service offers search results over a program interface for further computational analyses.

It is planned that CTIM will be able to support researchers by linking publications, gene data, and drug data with clinical trials data enabling cross-domain analysis. This advanced version of CTIM will link clinical trials data with related publications from PubMed or by finding biosamples data automatically. After querying the clinical trials database, the user will have the ability to search for related results in other biomedical databases.

## Status of development

Release candidate 1 (2014).

## Users

Scientists, clinicians.

## Link

CTIM Demonstrator [hhu3.at.xencon.de]; project site [http://www.biomedbridges.eu/]

